# Serum FSH Levels in Coasting Programmes on the hCG Day and Their Clinical Outcomes in IVF ± ICSI Cycles

**DOI:** 10.1155/2012/540681

**Published:** 2012-02-12

**Authors:** Srisailesh Vitthala, Jerome Bouaziz, Amanda Tozer, Ariel Zosmer, Talha Al-Shawaf

**Affiliations:** ^1^Kamineni Fertility Centre, Kamineni Hospitals, King Koti, Hyderabad 500001, India; ^2^Services of Gynecology and Obstetrics-Reproduction, The Bow Hospital, Chu de Nice, Route Saint Anton, 06200 Nice, France; ^3^Centre for Reproductive Medicine-Barts and The London NHS Trust, London EC 1A 7BE, UK

## Abstract

*Introduction*. Coasting is the most commonly used strategy in prevention of severe OHSS. Serum FSH levels measurements during coasting may aid in optimizing the duration of coasting. *Objective(s)*. To study live birth rates (LBRs), clinical pregnancy rates (CPRs), and optimal duration of coasting based on serum FSH levels on the hCG day. *Materials and Methods.* It is a retrospective study performed between 2005 and 2008 at Barts and The London Centre for Reproductive Medicine, NHS Trust, London, UK, on 349-coasted women undergoing controlled ovarian stimulation (COS) for IVF ± ICSI. The serum FSH level measurements on the hCG day during coasting programme were analysed to predict the LBR and CPR. *Result(s)*. LBR and CPR were significantly higher when the FSH levels on the hCG day were >2.5 IU/L (LBR: 32.5%, *P* = 0.045 and CPR: 36.9%, *P* = 0.027) compared to FSH <2.5 IU/L. The optimal FSH cut-off level for LBR and CPR is 5.6 IU/L on the hCG day. The optimal cutoff for coasting is 4 days. *Conclusion(s)*. Coasting may be continued as long as either serum FSH level is > 2.5 IU/L on the hCG day without compromising the LBR and CPR or to maximum of 4 days.

## 1. Introduction

Ovarian hyperstimulation syndrome (OHSS) is a rare iatrogenic complication of controlled ovarian stimulation (COS). It is one of the unpleasant complications of the gonadotrophin stimulation of the fertility treatment. The pathophysiology of OHSS is not fully understood, but it is hypothesized that clinical sequelae results in massive fluid shift in to the third space leading to intravascular dehydration. This is due to increased vascular permeability substances (VGEF and other growth factors). Although OHSS is self-limiting, it is the most serious complication of gonadotrophin stimulation with potentially serious and rarely fatal outcomes. Currently optimal classification of OHSS is based on clinical, biochemical, and sonographic parameters as follows described by Golan and Weissman.

Mild OHSS
Grade 1: Abdominal distention and discomfort.
Grade 2: Features of grade 1 plus nausea, vomiting, and or diarrhea. Enlarged ovaries (5–12 cm).
Moderate OHSS
Grade 3: Features of mild OHSS plus ultrasound evidence of ascites.
Severe OHSS
Grade 4: Features of moderate OHSS plus clinical evidence of ascites and or hydrothorax or breathing difficulties.
Grade 5: All of the above plus change in blood volume, increased viscosity due to haemoconcentration, coagulation abnormalities, and diminished renal perfusion and functions.
Grade 6: Severe or critically ill patients admitted in intensive care involving multiple organ systems.

To date, there is no known pharmacological agent that will cure OHSS. Hence, at best current treatment is empirical. Prevention or reducing the risk of OHSS still remains the best option [1–13].

Withholding hCG administration, resulting in treatment cancellation, remains the only method that will prevent the development of severe OHSS [8, 13]. However, treatment cancellation has significant financial and psychological implications. Henceforth, many strategies for reducing the risk of OHSS are tried. Coasting involves withdrawal of exogenous gonadotrophin stimulation, in high risk patients, once optimum follicular size has reached, and until the serum oestradiol levels drop to, what is considered to be a “safe” level to administer the hCG [4, 14, 15]. Coasting results in inhibition and apoptosis of granulosa cell proliferation due to falling levels of serum FSH and subsequently their luteinisation [14]. This will prevent or reduce the cascade of events that lead to severe OHSS [16].

Coasting is the most commonly used strategy in, prevention of severe OHSS. Even though evidence is not strong coasting played significant role in the past to prevent OHSS and it will be commonly employed in the future to prevent OHSS in the absence of clear understanding of OHSS pathophysiology. It also provides opportunity of pregnancy in the index cycle as well as freezing of surplus embryos and reduces the costs and the distress associated with the cycle cancellation [4, 14, 17–20]. Recent Cochrane systematic review reported benefit of coasting in reducing OHSS but no benefit in pregnancy rates when compared with no coasting [20].

Controversies still surrounds the coasting strategy regarding reduced number of oocytes retrieved, impaired implantation, and pregnancy rates. In particular, the optimal duration of coasting before hCG administration which will not affect pregnancy rates has not been elucidated [3, 15, 16, 21, 22].

The basis of serum FSH evaluation during coasting evolved from the understanding of physiology of follicular growth. Each follicle has a certain threshold level of FSH below which follicular growth does not occur [23–25]. An increase in the FSH level above the threshold level (after exogenous gonadotrophin stimulation) can induce many follicles to develop [24]. When serum FSH level falls below the threshold level of the follicle, then that particular follicle becomes susceptible to apoptosis, resulting in atresia. On this basis, the serum FSH levels should fall during coasting when gonadotrophins are withheld, and this in turn can influence ovarian response by decreasing the serum oestradiol levels [24, 26, 27]. Thus, monitoring falling serum FSH levels during coasting in addition to or alternative to serum oestradiol levels can aid to decide the number of days of coasting without compromising the outcomes.

Therefore, the aim of our study was to (1) investigate whether serum FSH levels on the hCG day would predict the clinical outcome in coasted women undergoing IVF ± ICSI cycles at risk of developing severe OHSS and (2) to find out the serum FSH level during coasting at which hCG can be administered safely without compromising the clinical outcomes. This will subsequently help to determine the optimal duration of coasting.

## 2. Materials and Methods

### 2.1. Study Design

This was a retrospective observational study conducted at Barts and The London Centre for Reproductive Medicine, NHS Trust, London, UK. Data from 349 women who were at risk of OHSS and were coasted for more than one day while undergoing controlled ovarian stimulation (COS) for IVF ± ICSI between 2005 and 2008.

### 2.2. Inclusion Criteria

Women who were at risk of OHSS identified and coasted for more than one day while undergoing controlled ovarian stimulation (COS) for IVF ± ICSI. The risk of OHSS women identified based on biochemical (serum E_2_ of >13000 pmol/L), clinical (previous OHSS, high risk patients as PCOS, <30 years, BMI <22, high response), and sonographic (≥20 follicles and >20% of them are ≥15 mm diameter) parameters during gonadotrophin stimulation. These women had their serum E_2_ and FSH levels measured from the day of coasting until the day of hCG administration on the hCG day and subsequently underwent oocyte retrieval fresh embryo transfer.

Ethical approval for this study was not required, since it only involved the analysis of recorded data on the unit's database. The mandatory informed consent obtained from all the women prior to the start of fertility treatment according the HFEA (fertility governing body in UK) allows the anonymous analysis of recorded data for clinical studies. All the women were clearly informed that their recorded data of fertility treatment may be used and analysed anonymously for clinical studies prior to the start of the treatment.

### 2.3. Treatment Protocol

The COS for IVF ± ICSI was downregulation with agonist protocol and gonadotrophin stimulation as previously reported [4]. In brief, the patients underwent long downregulation with a gonadotrophin releasing hormone (GnRH) agonist followed by gonadotrophin stimulation with the dose selected on the basis of age, previous response, early follicular phase serum FSH levels, and body mass index (BMI). Gonadotrophins were administered subcutaneously and the gonadotrophin used was urinary or recombinant dependent upon the availability and cost. The choice gonadotrophins did not influence outcomes in our unit over a decade. This is also supported by recent Cochrane systematic review [28]. Follicular monitoring was performed by transvaginal ultrasound, normally starting on day 9 of gonadotrophin stimulation but in some selected cases, when a high follicular response was anticipated (polycystic ovarian syndrome, previous high response), it is started on day 7 of stimulation.

Monitoring for the risk of OHSS was according to criteria published earlier [26]. In brief, serum FSH and E_2_ were measured when ultrasound scanning identified >20 follicles (>5 mm in mean diameter). When serum E_2_ was between 3000 and 13 200 pmol/L, and the leading follicles were >13 mm in mean diameter the gonadotrophin dose was halved. When serum E_2_ was >13 200 pmol/L and the leading follicles were >15 mm in mean diameter gonadotrophins were withheld hCG was administered when at least three follicles reached a mean diameter of ≥18 mm and serum E_2_ level was <10000 pmol/L. GnRH agonist was continued until the day of the hCG administration. Routine IVF ± ICSI laboratory procedures were used. The maximum numbers of embryos transferred were two in most of the cases and in very few women who were above 40 years of age had maximum of three embryos transferred. Vaginal progesterone pessaries were prescribed for luteal support. A urinary pregnancy test was performed 2 weeks following the embryo transfer and pregnant women had a transvaginal ultrasound scan 4 weeks later and all had confirmation of pregnancy outcome.

### 2.4. Hormonal Assay

The serum FSH was analysed using the Bayer immuno-1 automated analyser (Bayer, Newbury, Bucks, UK) calibrated to WHO second international reference preparation with a <5% coefficient of variation and inter- and intracoefficient of variation in the range of 1–25 IU/L and a sensitivity of 0.1 IU/L.

The serum E_2_ measurements were also performed by a Bayer immuno-1 automated analyser (Bayer, Newbury, Bucks, UK) with a <5% coefficient of variation in the range 75–13 200 pmol/L.

### 2.5. Outcomes

Clinical pregnancy rate (CPR) was defined as sonographic confirmation of an intrauterine gestation sac with a positive foetal heart beat. Live birth rate (LBR) was defined as the delivery of at least one viable baby.

The main outcomes analysed were LBR and CPR in relation to serum FSH level on the hCG day. The secondary outcomes analysed were the correlation between serum FSH level on the hCG day and age, BMI, basal FSH level, starting dose of gonadotrophin, duration of gonadotrophin administration, total dose of gonadotrophins administered, the number of oocytes retrieved, and the duration of coasting.

## 3. Statistical Analysis

The results were presented as means ± SD, percentages. The data was analyzed for LBR and CPR at five homogenous (Quintiles) serum FSH groups and also in (serum FSH ranging from ≤5.2, >5.2 to ≤7.4, >7.4 to ≤9.8, >9.8 to 12.8, >12.8 IU/L) on the day of hCG administration. This is to identify serum FSH levels at which LBR and CPR were significantly higher. Chi-square (*χ* ^2^) test was done as appropriate for comparison. *P* values of <0.05 were considered statistically significant.

The multivariate analyses were performed for the variables that could affect serum FSH levels on the hCG day after adjusting the confounders.

Receiver operating curve (ROC) analysis performed to identify the optimal serum FSH level (cut-off level) for hCG administration and also number days of coasting (duration of coasting) in coasted patients. All data were analyzed Stats Direct Statistical Software, version 2.7.7, London, UK.

## 4. Results

The demographic characteristics of the study population are described in Table 1. The mean serum FSH level on the hCG day was 9.8 ± 5.7 IU/L (range 1–38).

Eight patients (2.3%) had serum FSH levels <2.5 IU/L and 73(20.3%) patients had <5.2 IU/L on the day of hCG. The LBR and CPR were significantly higher when the FSH level was >2.5 IU/L than <2.5 IU/L on the day of hCG (LBR: 32.5%, *P* = 0.027 and CPR: 36.9%, *P* = 0.045) (Table 2).

**Figure 1:**
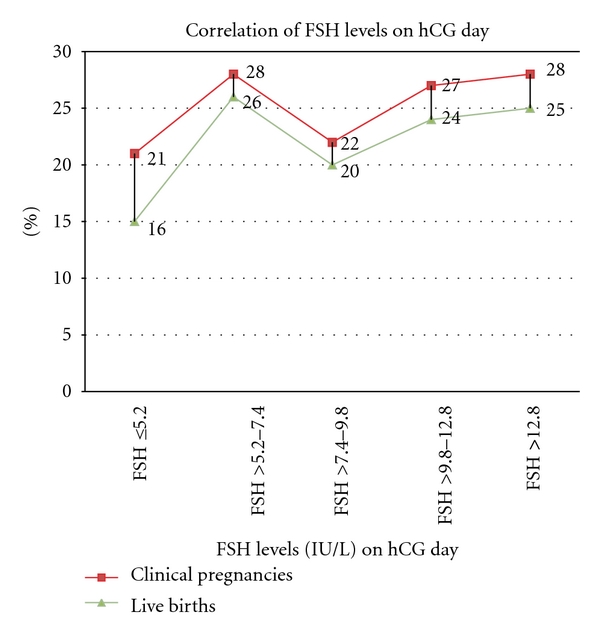


 The CPR and LBR were also calculated in five homogenous (Quintiles) groups of serum FSH levels (≤5.2, >5.2 to ≤7.4, >7.4 to ≤9.8, >9.8 to ≤12.8, >12.8 IU/L) on the day of hCG administration. Higher live birth rate was noticed only in the serum FSH group ranging >5.2 to ≤7.4 in comparison to ≤5.2 group but did not show any statistical difference between other groups (Table 2 and Figure 1).

Multivariate analysis showed that FSH levels on the day of hCG did not correlate with age, total dose of gonadotrophins administered, or the number of oocytes collected but showed a negative correlation with BMI (*P* < 0.0007), basal FSH (*P* = 0.0377) and the number of coasting days (*P* < 0.0001), and a positive correlation with the starting dose of gonadotrophins (*P* < 0.003). This can be translated to a single unit increase in BMI leads to a decrease in the serum FSH level by 0.2 IU/L on the day of hCG regardless of start dose, duration of coasting, and pretreatment basal FSH levels. Similarly, one unit increase in the level of pretreatment basal FSH leads to a decrease in the serum FSH level by 0.3 IU/L on the day of hCG regardless of the start dose, duration of coasting, and BMI.

**Figure 2:**
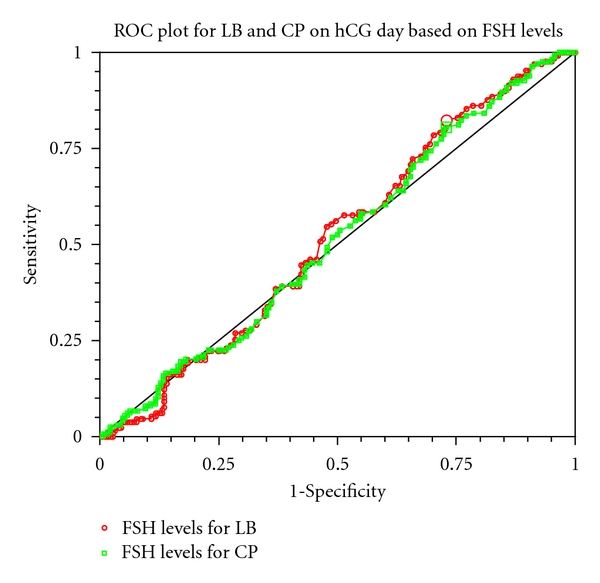
Live births (LB): optimum cut-off FSH value: 5.6; sensitivity: 78%; specificity: 27; ROC area under curve (AUC): 0.50 (95% CI: 0.70 to 0.85). Clinical pregnancies (CP): Optimum cut-off FSH value: 5.6; sensitivity: 80%; specificity 30%; ROC area under curve (AUC): 0.53 (95% CI: 0.73 to 0.85).

**Figure 3:**
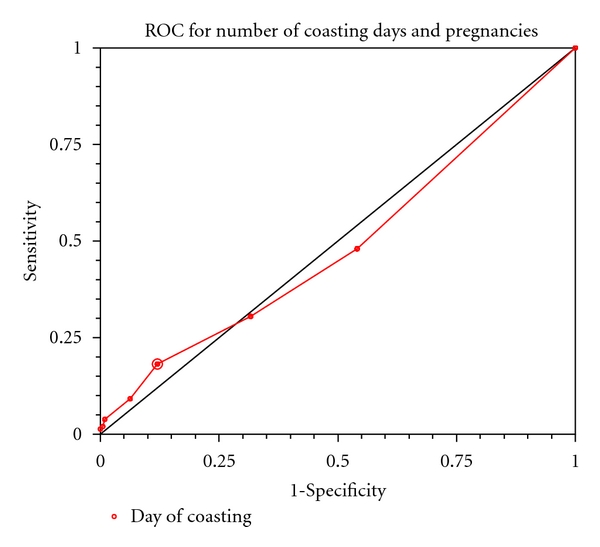
Optimum cut-off point selected: 4; area under ROC curve: 0.48; 95% CI: 0.45 to 0.56; sensitivity (95% CI): 0.18; specificity (95% CI): 0.88.

The optimum cut-off values for serum FSH levels on the hCG day for both live births (LB) and clinical pregnancies (CP) were 5.6 IU/L with a sensitivity of 78% and 80% and with a specificity of 27% and 30% respectively. This indicates that ROC curve did not predict absolute cut-off levels for LBR and CPR in this study (Figure 2). ROC area under curve (AUC) for live births (LB) and clinical pregnancies (CP) was 0.50 (95% CI: 0.70 to 0.85) and 0.53 (95% CI: 0.73 to 0.85), respectively. The optimum cut-off value for number days of coasting was 4 days with specificity of 88% and sensitivity of 12% indicating that chances of pregnancies after 4 days of coasting are very slim (Figure 3).

When we analyzed the number of oocytes retrieved by the number of days coasted, we noted that for each day of coasting the number of oocytes retrieved decreased by a factor of 0.6. The serum FSH declined 2.1 IU/L per day of coasting.

## 5. Discussion

This study reports the first large series to date on the correlation between LBR, CPR, and serum FSH levels on the hCG day. Results from our study suggests that serum FSH levels >2.5 IU/L on the day of hCG administration is associated with a clinically improved CP and LB than when serum FSH levels were <2.5 IU/L on the day hCG in women coasted to reduce risk of moderate/severe OHSS. Both CP and LB were significantly lower when FSH levels <2.5 IU/L on the day of hCG.

A recent study of 33-coasted cycles suggested an FSH cut-off level of 4.9 IU/L by ROC on the day of hCG for deciding the duration of coasting [29].They have reported no conceptions in cycles below their identified optimum FSH cut-off value (4.9 IU/L). We have found pregnancies with serum FSH level as low as 2.5 IU/L on the hCG administration day but not below the serum FSH level of <2.5 IU/L. Compared to them [27], ROC analysis in our study had the sensitivity at 80%, the specificity was low at 30%. The lack of good specificity to identify the absolute cut-off value in this study may be related to the difference in size of population compared. Moreover, it is difficult to compare and extrapolate our results (live births and clinical pregnancies) with their results (conceptions)[29]. It will be interesting to compare the similar outcomes in a larger population. The CPR and LBR were also calculated in five homogenous groups of serum FSH range levels (≤5.2, >5.2 to ≤7.4, >7.4 to ≤9.8, >9.8 to ≤12.8, >12.8 IU/L) on the day of hCG administration. Higher live birth rate was noticed only in the serum FSH group ranging >5.2 to ≤7.4 in comparison to ≤5.2 group but did not show a statistical difference between other groups.

Results from our earlier study has shown that serum FSH declines steadily (approximately 25% per day) during coasting, and the serum oestradiol will decline to a specific level of <10,000 pmol/L when FSH levels falls below the threshold level of 5 IU/L [25]. The optimum duration of coasting to administer hCG without compromising the pregnancy outcome may be decided on measurements of serum FSH in conjunction with daily measurements of serum oestradiol levels to ensure good clinical outcome patient safety and reducing the risk of severe OHSS. It is clinically useful to be aware that as serum FSH level declines and approaches levels ≤5 IU/L, careful decision making and monitoring will be required to decide on when to administer the hCG final injection in costing program.

Many authors have reported lack of correlation of serum E_2_ on hCG day and clinical outcome [13]. We did not include data on serum E_2_ levels and its relation to changes in serum FSH or correlation to clinical outcome in this study, as we intended to study the benefit of serum FSH measurement during the coasting strategy. Further analysis is in progress in our unit on this subject, where we intend to study correlation of serum FSH levels and serum E_2_ levels in coasting strategies. The identified serum FSH cut-off value of 5.6 IU/L, below which pregnancies do not occur in coasting programmes, needs to be further evaluated in large prospective, randomized controlled trials in view of low specificity.

 In this study, the serum FSH level decrease by 2.1 IU/L and the number of oocytes retrieved decrease by a factor of 0.6 with each day of coasting. Our results of oocyte retrieval with respect to duration of coasting were comparable to earlier studies [16, 29, 30]. Other studies in the literature reported significant reduction in number of oocytes retrieved after prolonged duration of coasting with different coasting strategies employed [13, 22]. The most probable explanation for reduced oocyte retrieval after prolonged coasting is due to poor follicular response (lack of LH receptor upregulation) to the exogenous hCG (trigger) subsequently leading to failure of final oocyte maturation. The oocytes that fail to undergo final maturation cannot be retrieved as they may stick to the follicular wall, hence, many follicles that are matured by size may not yield any oocytes [31].

The negative correlation of serum FSH level on the hCG day to the BMI and pretreatment basal FSH is difficult to explain but may be associated with factors like absorption, body fat distribution, and clearance [32]. The negative correlation of serum FSH on the hCG day on the duration of coasting is in agreement with other studies [22, 28, 33]. The duration of coasting will result in decreased serum FSH level which is approximately a drop of 25%/day or 2.1 IU/L [27]. The drop in the serum FSH levels leads to the apoptosis of granulosa cells during the coasting as explained in the introduction [22, 26, 27]. We did not find any studies in the literature reporting the effect of different variables on the serum FSH level on the hCG administration day.

More studies are required to evaluate the (a) efficacy of measuring serum FSH levels as a marker in coasting programmes in addition to serum E_2_ on the hCG day even if E_2_ is >10000 pmol/L and (b) percentage drop of serum FSH and oestradiol levels along with absolute cut-off values for the serum FSH and E_2_ levels with respect to CPR and LBR in coasted women to determine the optimum duration of coasting prior to hCG administration without compromising the outcomes.

We consider measuring serum FSH clinically beneficial to predict when serum E_2_ levels fall to safe levels and may aid in identifying the best value of serum FSH and E_2_ levels for best clinical outcome in coasting programme. More studies are needed to evaluate the value of measuring serum FSH in coasted women alongside in with serum E_2_ to determine the optimal duration of coasting or optimal time to administer hCG without compromising the clinical outcomes in coasting programmes. Further studies will assist in determining the value of measuring serum FSH in COS in general.

## 6. Conclusion

We conclude that in this study we noticed that LBR and CPR were significantly higher when serum FSH level was >2.5 IU/L on the hCG day. Our study also suggests LBR and CPR may not compromise as along as serum FSH levels do not fall below <2.5 IU/L on the hCG day. Hence, we suggest serum FSH monitoring during coasting programmes may provide reassurance of efficacy coasting programmes.

## Figures and Tables

**Table 1:** Demographic characteristics and variables of the study population.

*n* = 349	Mean ± SD (range)
Age (years)	33.0 ± 4.2 (21–44)
Body mass index (kg/m^2^)	24.7 ± 4.5 (18–43)
Basal (day 1-2) FSH (IU/L)	6.6 ± 2.0 (1.9–15)
Gonadotrophin start dose (IU)	211.5 ± 78.0 (100–450)
Gonadotrophin total dose (IU)	1989 ± 1139 (450–13500)
FSH on hCG administration day (IU/L)	9.8 ± 5.7 (1–38)
Number of coasted days	2.08 ± 1.2 (2–7)
Number of eggs collected	13.13 ± 5.1 (2–38)

**Table:** (a) CPR and LBR at FSH levels <2.5 IU/L and >2.5 IU/L

FSH levels at HCG trigger (IU/L)	Number (%) *n* = 349	Clinical pregnancy rate CPR (%)	Live birth rate LBR (%)
<2.5	8(2.3)	0	0
>2.5	341 (97.7)	126 (36.9)	111 (32.5)
Total	349	*P* = 0.027* significant	*P* = 0.045* significant

**Table:** (b) CPR and LBR at different (Quintiles) FSH levels in IU/L

FSH	Number (%)	CPR (%)	*P* value	LBR (%)	*P* value
FSH ≤5.2	73 (20.9)	21 (28.7)	0.09	16 (21.6)	0.02* significant
FSH >5.2–7.4	69 (19.7)	28 (40.5)		26 (37.6)	
FSH >7.4–9.8	68 (19.4)	22 (32.3)	0.20	20 (29.4)	0.20
FSH >9.8–12.8	70 (20)	27 (38.5)	0.27	24 (34.2)	0.33
FSH >12.8	69 (19.7)	28 (40.5)	0.47	25 (36.2)	0.47
